# Feasibility of a rat femoral-defect model for immediate perioperative and delayed post-implantation implant-associated bone infections

**DOI:** 10.5194/jbji-11-535-2026

**Published:** 2026-08-25

**Authors:** Pardis Keikhosravani, Michiel Croes, Nada Ristya Rahmani, Flurina Stabuli, Leonardo Cecotto, H. Charls Vogely, Bart C. H. van der Wal, Marianne Koolen, Debby Gawlitta, Harrie Weinans, Azin Khodaei, Saber Amin Yavari

**Affiliations:** 1 Department of Orthopedics, University Medical Center Utrecht, Utrecht 3508GA, the Netherlands; 2 Department of Maxillofacial Surgery, University Medical Center Utrecht, Utrecht 3508GA, the Netherlands; 3 Department of Biomechanical Engineering, Delft University of Technology, 2628 CD, Delft, the Netherlands; 4 Regenerative Medicine Centre Utrecht, Utrecht University, 3508 GA Utrecht, the Netherlands

## Abstract

**Background**: Implant-associated bone infections (IAIs) represent a major clinical challenge, causing implant failure, prolonged morbidity, and costly revision surgeries. Infection risk is highest in the immediate perioperative period, but delayed post-implantation contamination, occurring weeks after surgery in a partially healed host, represents an equally important and underappreciated clinical scenario. Bacteria rapidly colonize implant surfaces and form biofilms that resist both antibiotics and host immune responses, making prevention critical. Well-characterized small-animal models that capture both early and delayed post-implantation infections are therefore essential to develop and test antibacterial implants and coatings. **Methods**: We developed a rat femoral-segmental-defect model stabilized with a polyether-ether-ketone plate and a 3D-printed porous titanium implant. Three studies were performed using a total of 28 animals (3 excluded due to early humane endpoints; n=25 in final analysis): (i) early inoculation during surgery with planktonic *Staphylococcus aureus* (ATCC 49230) at 10^4^ or 10^6^ CFU (colony-forming units) per rat, (ii) delayed inoculation 28 d after surgery with planktonic *S. aureus* at 10^4^ or 10^6^ CFU per rat, and (iii) delayed inoculation with 10^8^ CFU per rat delivered as planktonic or ruptured-biofilm inoculum. Controls received phosphate-buffered saline at implantation. The primary endpoint was infection at day 14, quantified by CFU enumeration from homogenized bone and from a sonicated implant, plate, and screws. Micro-computed tomography (micro-CT) was used to visualize fixation and hardware position. **Results**: Early inoculation with 10^4^ or 10^6^ CFU per rat produced consistent infections across bone, implants, and screws. The same doses given 28 d later yielded low and inconsistent colonization. Escalation to 10^8^ CFU per rat in the delayed setting produced consistent infections; the ruptured-biofilm inoculum generated higher implant-associated bacterial burdens than planktonic suspensions, while bone burdens were consistently high in both arms. **Conclusions**: This femoral-implant rat model demonstrates feasibility for studying both immediate perioperative and delayed post-implantation infections, providing a platform for preclinical evaluation of anti-infection strategies. Timing, inoculum magnitude, and bacterial state critically determine infection establishment.

## 1 Introduction

Implant-associated infections (IAIs) are among the most severe complications in orthopedic surgeries, especially in procedures involving joint prostheses and internal fixation devices (Fang et al., 2017; Witso, 2014). Bacteria can adhere to biomaterial surfaces and develop structured biofilms that are markedly more tolerant to antibiotics and host immune responses (Arciola et al., 2018; Schömig and Putzier, 2020; Zimmerli et al., 2004). As a result, IAIs frequently become chronic, delay bone healing, and ultimately require revision surgery or implant removal (Bakalakos et al., 2024; Gatti et al., 2022). Among the causative organisms, *Staphylococcus aureus* (*S. aureus*) is particularly problematic, as it readily adheres to implant surfaces and establishes persistent biofilms (Gatti et al., 2022; Gbejuade et al., 2015).

Bacteria begin to colonize the implant surface shortly after surgery and switch to a biofilm state, so biofilm formation is relevant in both early- and later-presenting infections, while factors such as timing of contamination, local tissue environment, and host immunity determine whether disease manifests as an acute or a chronic presentation. Understanding both infection types is crucial for designing preventative strategies and testing long-acting antibacterial coatings or systemic treatments (Gjini et al., 2020; Jahanmard et al., 2020). Biofilm-associated infections prolong recovery times and impose substantial economic and healthcare burdens globally (Assefa and Amare, 2022).

In recent years, antibacterial biomaterials, such as antibiotic-releasing or biofilm-resistant coatings, have emerged as a promising strategy to prevent IAIs (Amin Yavari et al., 2020). However, successful clinical translation of these materials depends on reliable preclinical evaluation in physiologically relevant in vivo models (Frisch et al., 2023). Existing infection models, although commonly used, vary considerably in construct design, inoculation timing, and bacterial phenotype, limiting direct cross-study comparison. While rodent models investigating delayed-infection susceptibility or staged surgical approaches have been described, a model combining a defined femoral segmental defect with a structured fixation construct, multiple inoculation windows, and phenotypic comparison of bacterial state within a single framework has not been systematically established (Guarch-Pérez et al., 2021; Huang et al., 2023; Seebach and Kubatzky, 2019). This gap limits clinical predictability and delays effective antibacterial solutions.

Existing orthopedic infection models already span intramedullary pin or wire systems, metaphyseal screw models, knee prosthesis constructs, and staged non-union or revision models, but systematic reviews emphasize major heterogeneity in species, implant design, bacterial strain, inoculation route, inoculum size, and outcome measures, which complicates direct cross-study comparison rather than indicating that comparable models are absent (Guarch-Pérez et al., 2021; Helbig et al., 2020; Moriarty et al., 2019; Reizner et al., 2014). Dose-response behavior is strongly construct-dependent: in rat prosthetic models, 10^2^ CFU (colony-forming units) may be insufficient, 10^3^ CFU can establish osteomyelitis without implant loosening, and ≥ 10^4^ CFU can induce more destructive infection, whereas other metaphyseal screw models have achieved consistent infection with only 10^2^–10^3^ CFU (Harrasser et al., 2016; Soe et al., 2013). Bacterial phenotype is also emerging as an important design variable, as biofilm-based inocula and low-metabolic bacterial aggregates can produce different infection rates and tissue responses from conventional planktonic challenges (Shaw et al., 2023; Top Hartmann et al., 2024). Within this landscape, the value of the present work lies in comparing inoculation timing, dose, and bacterial phenotype within a single healed femoral-segmental-defect construct relevant to antibacterial implant testing.

To address this, there is a growing demand for standardized animal models that reliably simulate the complexities of both early (immediately post-surgical) and delayed IAIs. The delayed scenario modeled here (4 weeks post-implantation) is intended to reflect delayed surgical site contamination or early hematogenous seeding in a partially healed host environment, rather than chronic established infection (Chen et al., 2025; Steinmetz et al., 2019). Current models often vary in terms of bacterial strain, inoculum dose, infection timing, and anatomical site, complicating cross-study comparison and limiting standardization (Chen et al., 2025; Helbig et al., 2020; Moriarty et al., 2019). Moreover, challenges such as host–pathogen variability, ethical concerns, species-specific immune responses, or bacterial state (biofilm or planktonic) further complicate model design and interpretation (Holdbrook et al., 2025; Li and Baldridge, 2023; Zimmerli and Sendi, 2019). A refined in vivo model that accounts for these complexities is essential to bridge the gap between laboratory findings and clinical application. The ideal preclinical model must enable controlled induction of infection while preserving animal welfare and allowing precise assessment of therapeutic efficacy (Chen et al., 2025; Moriarty et al., 2019). It should allow interrogation of different clinically relevant contamination windows and bacterial states (planktonic versus biofilm-derived); facilitate the testing of long-acting antibacterial coatings; and provide reproducible, quantifiable outcomes (Giavaresi et al., 2014; Shi et al., 2018).

In this study, we developed a reproducible rat femoral-defect model designed to capture both early perioperative and delayed post-implantation bacterial challenges at the implant site. Immediate infection was modeled by inoculating the defect at the time of implantation, whereas post-implantation contamination was simulated by inoculation 4 weeks after implantation using either planktonic bacteria or disrupted biofilm. By varying inoculum timing, dose, and bacterial state, we identified conditions that yield consistent infection burdens while respecting humane endpoints. This dual-time-point infection model provides a clinically relevant platform for testing antibacterial biomaterials under perioperative and later post-implantation infection scenarios.

## 2 Materials and methods

### 2.1 Experimental design

#### 2.1.1 Animals and surgical procedure

Adult male Wistar rats 12 weeks in age (skeletally mature with closed growth plates, ensuring stable fixation and consistent bone quality relevant to infection susceptibility) were obtained from Charles River (L'Arbresle, France) and housed in pairs at the Central Laboratory Animal Institute (Utrecht University) under controlled environmental conditions (21 °C, 12 h light–12 h dark cycle), with ad libitum access to standard rodent chow and water. Animals underwent a 7 d acclimatization period prior to surgery, and health status was monitored daily.

Surgical procedures were performed under general anesthesia with 1 %–3.5 % isoflurane. Preoperative analgesia consisted of buprenorphine (0.03 mg kg^−1^, Temgesic^®^, RB Pharmaceuticals Limited, UK) administered once before surgery. The surgical site was shaved and disinfected with povidone–iodine. A 6 cm lateral skin incision was made to expose the right femur, followed by division of the fascia. A 23 mm polyether-ether-ketone (PEEK) fixation plate (RISystem, Switzerland) was applied to the anterolateral femur using six screws. The periosteum was removed over 8 mm of the mid-diaphysis, and a 6 mm cortical segmental defect was created using a saw guide and wire saw (RISystem, Switzerland). A 3D-printed porous Ti implant was press-fit into the defect, and the fascia and skin were closed using resorbable VICRYL Rapide 4-0 sutures (Ethicon, US). Additional postoperative buprenorphine was administered twice daily for 3 d. Body weight was recorded regularly throughout the study period. Animals were monitored daily for signs of systemic illness (lethargy, labored breathing, hunched posture), wound dehiscence, and local complications. Predefined humane endpoints included > 20 % body weight loss, severe wound breakdown, or clinical signs of systemic sepsis; animals meeting these criteria were euthanized early and their data excluded from analysis.

#### 2.1.2 Experimental groups (early and delayed models)

Seven groups (each n=4 ) were investigated in three experimental settings (total n=28 ). Three animals were lost before the planned study endpoint and excluded from the final analysis (one each from the D-P-10^4^, D-P-10^6^, and D-P-10^8^ groups, reducing those group sizes from n=4 to n=3 ): two died due to anesthesia-related surgical complications, and one was euthanized due to implant dislodgement from the femoral defect postoperatively, which compromised construct integrity. Data from these animals were excluded from the final analysis. One negative control group received phosphate-buffered saline (PBS) immediately after implantation.

#### Early-infection model (Experiment 1) 

Immediately after implantation, two groups received a local inoculation of *S. aureus* directly onto the implant surface: 10^4^ CFU in 500 µL ( n=4 ) or 10^6^ CFU in 500 µL ( n=4 ). Animals were euthanized with CO_2_ 14 d post-inoculation, and femora with implants were retrieved for CFU quantification and micro-computed tomography (micro-CT; Quantum FX, PerkinElmer, USA).

#### Delayed-infection model – planktonic (Experiment 2)

At 28 d post-implantation, animals were anesthetized with isoflurane, and *S. aureus* was injected percutaneously into the implant site: 10^4^ CFU in 500 µL ( n=3 ) or 10^6^ CFU in 500 µL ( n=3 ). Euthanasia and sample collection were performed 14 d later as above.

#### Delayed-infection model – planktonic vs. ruptured biofilm (Experiment 3)

At 28 d post-implantation, animals were anesthetized with isoflurane and received buprenorphine analgesia as for the initial surgery. The right hind limb was shaved and disinfected, and a ∼ 4 cm incision was made to re-expose the implant. Two groups were inoculated directly onto the implant with 10^8^ CFU in 500 µL : planktonic ( n=3 ) or ruptured-biofilm phenotype ( n=4 ). The incision was closed, and postoperative care was identical to that described for the initial surgery. Open re-exposure was selected for Experiment 3 to enable accurate direct inoculation onto the implant surface at the higher bacterial dose; while this approach disrupts the healed peri-implant tissue and may transiently reduce local host defenses compared to percutaneous delivery used in Experiment 2, it ensures reliable inoculum placement. This methodological difference is acknowledged as a potential confounding variable when comparing infection outcomes across experiments. Euthanasia and sample collection were performed 14 d later.

**Table 1:** Standardized experimental group naming table.

Abbreviation	Infection model	Bacterial state	Inoculation dose	Inoculation timing
CTRL	No infection (control)	–	–	Day 0 (PBS only)
E-P-10^4^	Early infection	Planktonic	10^4^ CFU per rat	Day 0
E-P-10^6^	Early infection	Planktonic	10^6^ CFU per rat	Day 0
D-P-10^4^	Delayed infection	Planktonic	10^4^ CFU per rat	Day 28 (percutaneous)
D-P-10^6^	Delayed infection	Planktonic	10^6^ CFU per rat	Day 28 (percutaneous)
D-P-10^8^	Delayed infection	Planktonic	10^8^ CFU per rat	Day 28 (open surgery)
D-B-10^8^	Delayed infection	Ruptured biofilm	10^8^ CFU per rat	Day 28 (open surgery)

### 2.2 Bacterial culture

The bacterial strain used in all experiments was *S. aureus* ATCC 49230 (American Type Culture Collection, Manassas, VA, USA). This clinical isolate, originally obtained from a patient with chronic osteomyelitis, has previously been demonstrated to reliably establish persistent bone infections in rat models using an intramedullary K-wire. Inoculum doses of 10^4^ and 10^6^ CFU per rat were selected based on published rat implant infection models reporting consistent early infection at these ranges; the escalation to 10^8^ CFU per rat in the delayed setting was empirical, applied after lower doses failed to produce reliable colonization at 28 d post-implantation (Croes et al., 2018; Lucke et al., 2003).

For the “planktonic inoculation”, overnight cultures were grown in tryptic soy broth (TSB) at 37 °C with shaking. On the day of inoculation, cells were pelleted (17 000 ×g , 2 min, room temperature), the supernatant was discarded, and the pellet was resuspended in sterile PBS; this wash was repeated twice to remove residual growth medium that could favor bacterial growth after injection. Suspensions were then adjusted to 10^4^, 10^6^, or 10^8^ CFU per rat (see Table 1) in sterile PBS for direct in vivo injection. The planktonic inoculum therefore consists of stationary-phase bacteria, freely suspended, non-adherent cells with planktonic physiological characteristics and relatively higher antibiotic susceptibility compared to biofilm-embedded cells.

For the “ruptured-biofilm inoculation” (D-B-10^8^; Table 1), biofilms were generated from a previously established protocol (Keikhosravani et al., 2025). Briefly, an overnight bacterial culture was diluted 1:100 in fresh TSB and incubated at 37 °C, shaking until mid-log phase (optical density (OD) at 600 nm of 0.5–0.6). The culture was then diluted to OD ${}_{600}=0.1$ in TSB supplemented with 0.5 % glucose and 3 % NaCl to promote biofilm formation. Aliquots of 2 mL were dispensed into 24-well plates and incubated at 37 °C under mild shaking (150–200 rpm) for 48 h. After 24 h, half of the culture medium was gently replaced with fresh medium, taking care not to disrupt the biofilm structure. After incubation, the medium was removed, and wells were gently washed twice with PBS to eliminate non-adherent bacteria. The biofilm was then carefully scraped from the well surface using a sterile loop or pipette tip and transferred to a sterile tube. To prepare ruptured-biofilm suspensions, the harvested biofilm was sonicated for 10 min to disperse bacterial aggregates. It should be noted that sonication disrupts the extracellular polymeric substance (EPS) matrix and may alter bacterial surface physiology; the inoculum therefore represents a disrupted biofilm state rather than intact biofilm fragments. Quantification was performed using CFU assay.

### 2.3 Micro-CT scanning

Micro-CT imaging was performed post-mortem with acquisition parameters set at 90 kV tube voltage, 180 µA tube current, and a 30 mm field of view. The scans were reconstructed into a series of 2D TIFF slices with an isotropic voxel size of 60 µm . Image analysis was conducted using the BoneJ plugin (version 1.3.12) within ImageJ software (version 1.48; National Institutes of Health, USA). Qualitative assessment focused on confirming hardware positioning and implant integrity across different angles.

### 2.4 Determination of bacterial load

All procedures were performed under sterile conditions. Harvested femora were homogenized in 5 mL of PBS using a homogenizer (Polytron PT3100, Kinetica Benelux, the Netherlands). Homogenated bones were serially diluted in PBS, plated in triplicate on Todd–Hewitt agar plates, and incubated overnight at 37 °C to allow colony formation. The day after, CFUs were counted to quantify the bacterial burden. Contralateral, non-operated femora served as negative controls and consistently showed no bacterial growth, confirming that infection was localized to the inoculated defect site.

Following bone processing, retrieved implants, plates, and screws were rinsed three times with PBS to remove loosely adherent material; wash fractions were not plated separately, as the primary aim was to quantify firmly surface-adherent bacteria via sonication. The implants, plates, and screws were then separately sonicated in PBS for 1 min to detach surface-adherent bacteria. The resulting sonicate was serially diluted and plated for CFU count as described above. Future studies should consider plating wash fractions to enable distinction between loosely adherent and firmly attached bacterial populations.

### 2.5 Statistical analysis

Individual animals were considered the experimental unit. Group sizes were control (CTRL, n=4 ), early infection of 10^4^ CFU per rat (E-P-10^4^, n=4 ), early infection of 10^6^ CFU per rat (E-P-10^6^, n=4 ), delayed infection of 10^4^ CFU per rat (D-P-10^4^, n=3 ), delayed infection of 10^6^ CFU per rat (D-P-10^6^, n=3 ), optimized delayed infection of 10^8^ CFU per rat planktonic (D-P-10^8^, n=3 ), and optimized delayed infection of 10^8^ CFU per rat biofilm (D-B-10^8^, n=4 ). Data are presented as mean ± SD unless stated otherwise. Given the small group sizes ( n=3 –4) and likely non-normal distribution of CFU data, formal inferential statistics were not applied; all comparisons should be interpreted as descriptive trends. CFU values of zero were included as observed. Infection take rate (proportion of animals with detectable CFU > 0) is reported per group to supplement mean values and provide clearer insight into consistency of infection establishment. Future studies with adequately powered cohorts should apply non-parametric tests (e.g., Mann–Whitney U ) with correction for multiple comparisons.

## 3 Results

### 3.1 Surgical feasibility and postoperative observations

**Figure 1:**
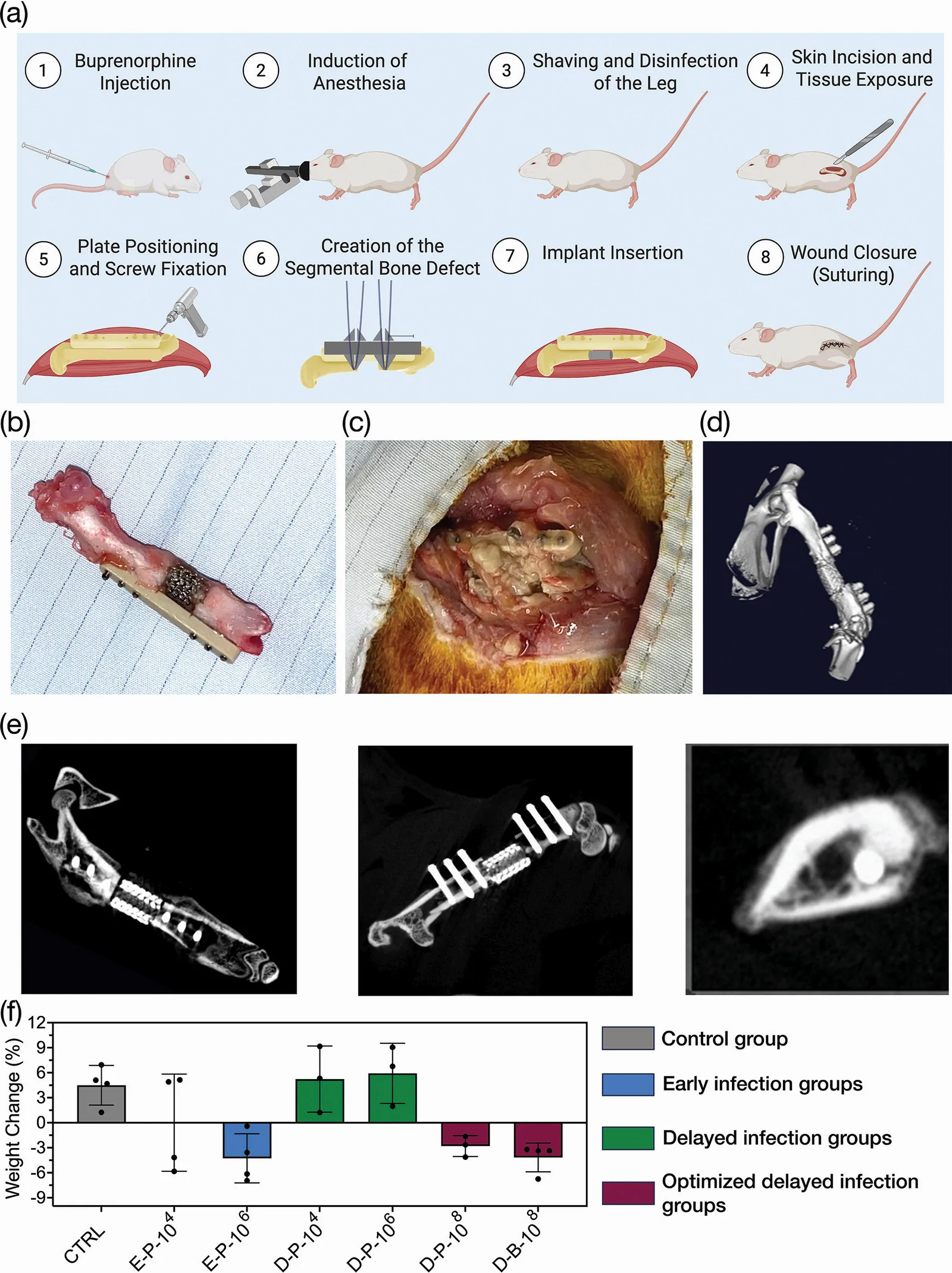
Establishment and characterization of a rat femoral-implant-infection model. **(a)** Schematic representation of the surgical steps for segmental-femoral-defect creation and titanium implant placement in rats (illustrated by http://biorender.com/). **(b)** Image of an explanted femur with the titanium implant and PEEK plate. **(c)** Intraoperative image showing local infection signs at the defect site prior to explantation. **(d)** Representative micro-CT reconstruction (3D) illustrating implant position and hardware configuration. **(e)** Micro-CT reconstructions of femora with titanium implants across various angles. **(f)** Quantitative analysis of body weight change (%) from baseline to endpoint across all experimental groups, including uninfected controls (CTRL), early-infection groups (E-P-10^4^, E-P-10^6^), delayed-infection groups (D-P-10^4^, D-P-10^6^), and optimized delayed-infection groups (D-P-10^8^, D-B-10^8^).

Post-mortem photographs confirmed a stable construct with the porous Ti segment press-fit within a 6 mm femoral defect and fixed by a PEEK plate and screws (Fig. 1b). The dissected defect region exhibited purulent material and necrotic debris at infected sites (observed in 8 of 11 infected animals across Experiments 1–3) (Fig. 1c, post-mortem field view). Micro-CT was used to visualize implant fixation and hardware positioning from different angles (Fig. 1d and e).

Across the study, body weight remained broadly stable, with group-specific trends. Controls exhibited a modest gain of 2 % to 4 %. Early-infection animals showed the greatest variability, with the higher-inoculum group tending toward mild weight loss from - 2 % to - 4%. Animals in the initial delayed-infection groups (10^4^–10^6^ CFU per rat) generally maintained their starting weight, showing modest gains of approximately 1 % to 6 %. In the delayed-infection study (10^8^ CFU per rat), both planktonic and ruptured-biofilm groups showed weight losses typically within - 2 % to - 5 % (Fig. 1f).

### 3.2 Experiments 1 and 2: bacterial burden of an early- and delayed-infection model

#### 3.2.1 Early infection (day 0 inoculation, day 14 harvest)

Both early-infection groups yielded consistent bacterial recovery across all sampled compartments (E-P-10^4^ and E-P-10^6^ groups). After 14 d the bones showed similarly high bacterial loads for both groups at 10^5^ to 10^6^ CFU per sample. Implants showed consistent colonization at roughly 10^3^ to 10^4^ CFU per sample. Plates and screws demonstrated considerable bacterial load at about 10^5^ CFU per sample. No growth was detected in uninfected controls (PBS injection). 

#### 3.2.2 Delayed infection (day 28 inoculation, day 42 harvest)

In contrast, delayed inoculation with 10^4^ to 10^6^ CFU of planktonic *S. aureus* per rat resulted in low and inconsistent colonization (D-P-10^4^ and D-P-10^6^ groups). Bone cultures were sporadically positive in the D-P-10^4^ group, with typically ∼ 10^3^ CFU per sample 2 weeks after inoculation, and remained low in the D-P-10^6^ inoculated group. Implant samples were culture-negative in all animals in both the D-P-10^4^ and D-P-10^6^ groups (take rate: 0/3 in both groups), indicating complete absence of detectable implant colonization at these doses. For plates and screws, the take rate was similarly 0/3 in the D-P-10^4^ group; in the D-P-10^6^ group, only one out of three animals showed any detectable CFU, with values close to the detection limit (Fig. 3b). These findings show that the D-P-10^4^ and D-P-10^6^ groups did not reliably establish a delayed infection at the implant site.

**Figure 2:**
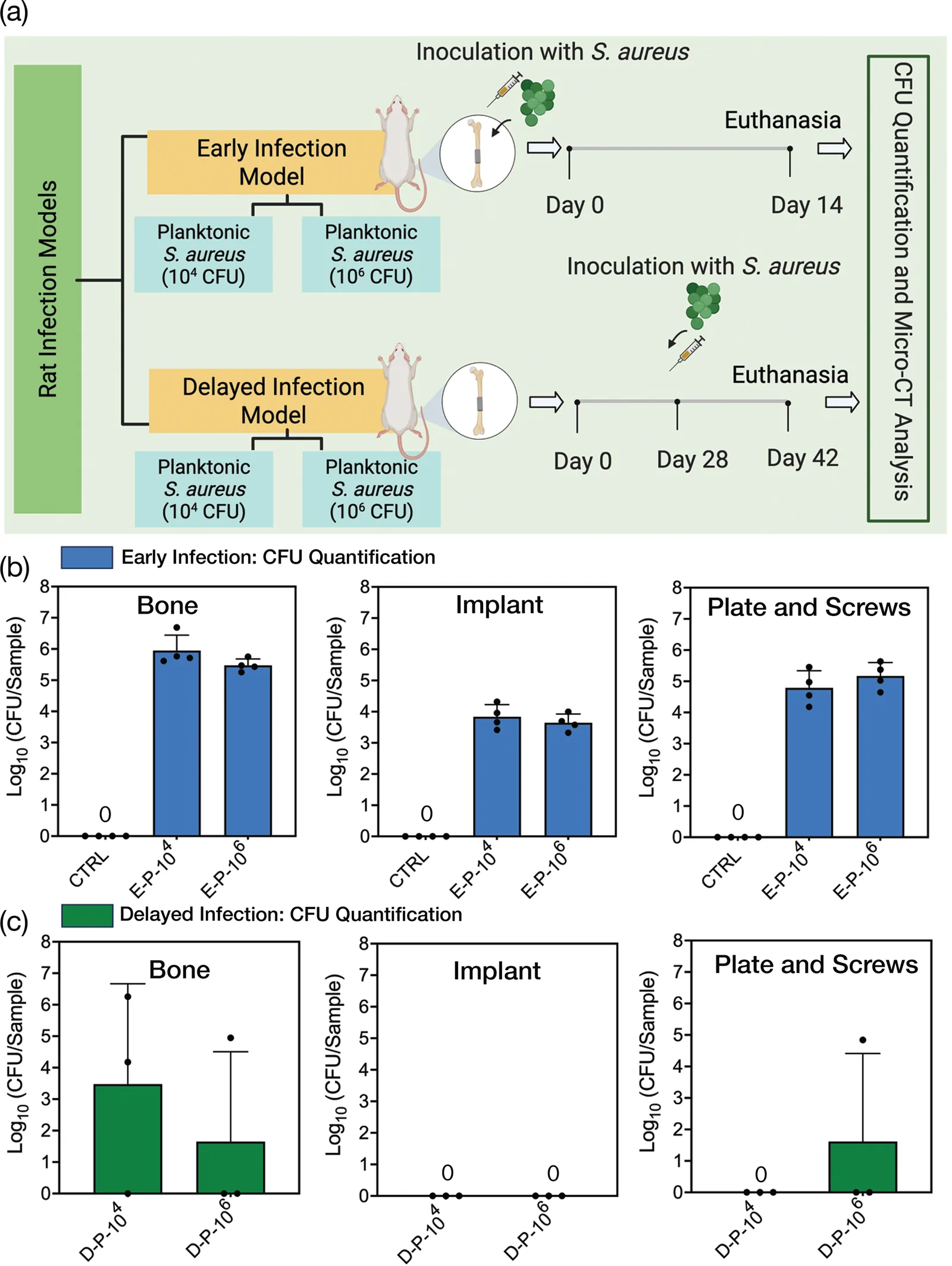
Experimental design and bacterial burden in early- and delayed-planktonic-infection models (Experiments 1 and 2). **(a)** Timeline overview for Experiments 1 and 2, outlining the key events including the surgery, bacterial inoculation, and eventual euthanasia of the rats in the study (illustrated by http://biorender.com/). Colony-forming unit (CFU) results for the **(b)** early- and **(c)** delayed-infection models, demonstrating the bacterial load at varying inoculation doses (10^4^ and 10^6^ CFU) in bone, implants, plates, and screws.

### 3.3 Experiment 3: bacterial burden with higher inoculation and biofilm

The site was re-opened at day 28 and inoculated with 10^8^ CFU per rat as either a planktonic suspension (like Experiment 2) or ruptured biofilm (D-P-10^8^ and D-B-10^8^ groups). By day 42, both groups showed sustained bone infection, with detectable bacterial loads in all animals, indicating detectable colonization at this inoculum dose. At the device interface, ruptured biofilm produced higher CFU (descriptive trend; no inferential statistics applied) than the planktonic state. Sonicated plates and screws were positive in both groups, with a tendency toward higher values after biofilm-derived inoculation.

**Figure 3:**
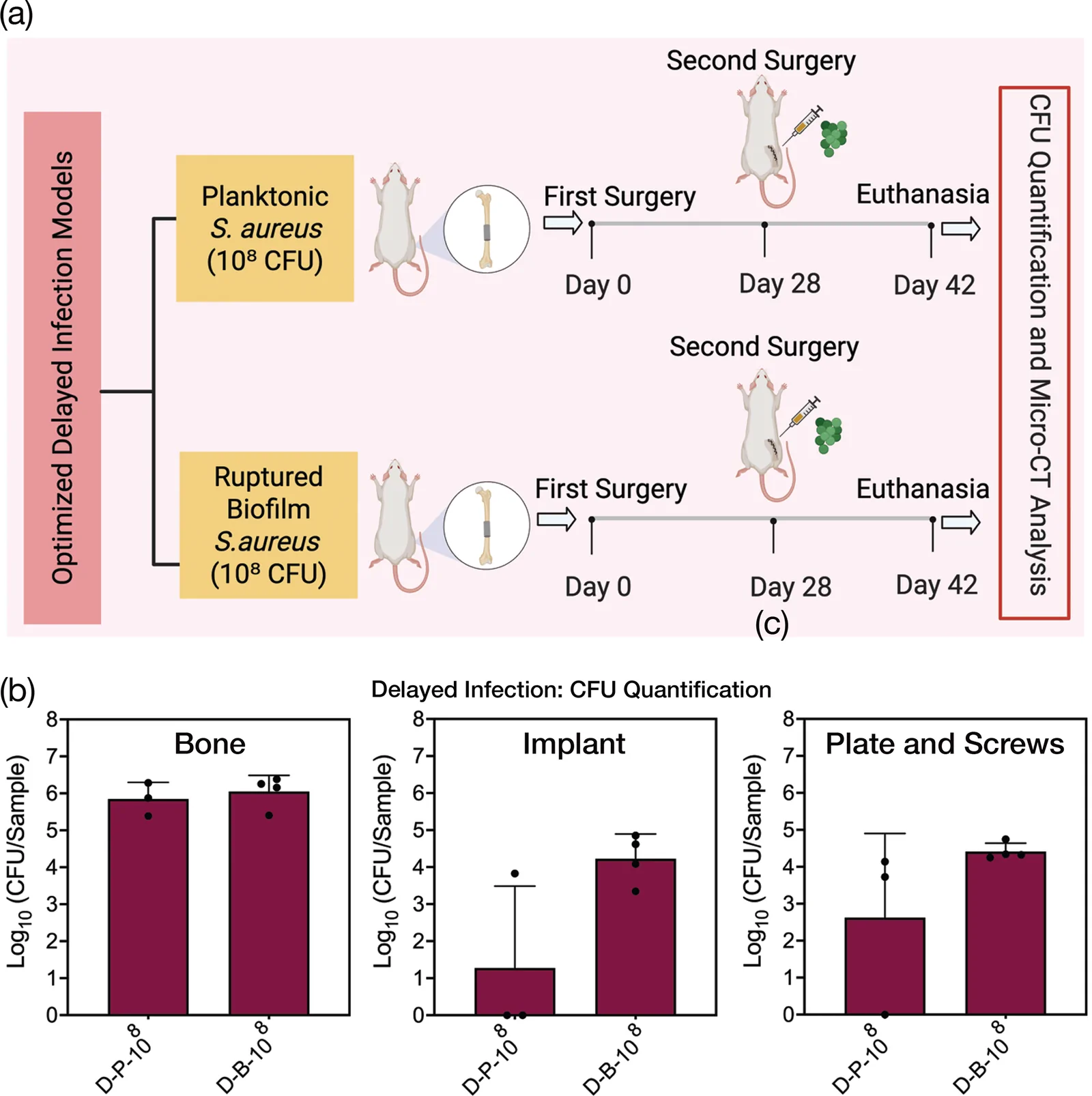
Delayed-infection model comparing planktonic and biofilm inoculate (Experiment 3). **(a)** Timeline schematic for Experiment 3, illustrating the sequence of surgery, bacterial inoculation, and subsequent euthanasia of rats, specifically for investigating the delayed post-implantation (illustrated by http://biorender.com/). **(b) **Colony-forming unit (CFU) results for the delayed infection in Experiment 3, comparing between two different states of *S. aureus*: planktonic and ruptured biofilm with 10^8^ CFU in bone, implants, plates, and screws.

## 4 Discussion

In this rat femoral-defect model, we established *S. aureus* contamination at two clinically relevant windows: immediately perioperative and delayed post-implantation. Early infections were reliably induced with 10^4^ and 10^6^ CFU of planktonic bacteria per rat delivered during surgery, whereas delayed infection (4 weeks after surgery) required a higher inoculum (10^8^ CFU per rat) to yield consistent colonization. In the delayed setting, a ruptured-biofilm inoculum produced higher implant/plate/screw colonization than an equivalent planktonic dose, while bone burdens were similar. Together, these data confirm the feasibility of a dual-time-point infection model and show that timing and bacterial phenotype distinctly shape infection within the same implant–host construct over time.

Compared to simpler implant infection models, such as intramedullary K-wire or transcortical screw systems, the segmental defect construct introduces a larger implant–tissue interface, a structured porous scaffold surface, and a more complex mechanical environment, all of which more closely reflect the clinical scenarios of tumor resection, severe trauma, or revision surgery where infection risk is highest. While these simpler models are appropriate for studying basic infection biology, the present construct was specifically designed to serve as a platform for future evaluation of antibacterial coatings applied directly to the porous Ti scaffold. The bacteriological characterization reported here is therefore a prerequisite step, establishing infection conditions within this construct before therapeutic evaluation is undertaken(Helbig et al., 2020; Moriarty et al., 2019).

The porous Ti implant was selected specifically because its open-pore architecture provides a structured surface for bacterial adhesion that closely mirrors the geometry of clinically used porous implants, such as those used in load-bearing bone reconstruction. Furthermore, the critical-size segmental defect, rather than a simpler transcortical or intramedullary model, was chosen to replicate the mechanical and biological environment of large-segment fixation constructs, which carry a disproportionately high infection risk clinically, and to enable future direct evaluation of antibacterial coatings on the same standardized construct.

We used male, skeletally mature rats (sex was controlled to minimize immune variability; estrogen-mediated immune differences may influence infection susceptibility independently of inoculation parameters) for surgical feasibility and to reduce hormone-related variability in bone healing; estrogen cycling can introduce heterogeneity in healing outcomes. Maturity ensured stable fixation and minimized growth-related remodeling (Liu et al., 2019). The dose gap between early and delayed models underscores how the host environment and timing determine susceptibility (Bahnasawy et al., 2025; Monaco et al., 2017). During surgery, even doses as low as 10^4^ CFU seeded infection, likely because the implant surface is fresh, the tissue has been disrupted, and the acute inflammatory response has not fully contained contaminants (de Aguilar-Nascimento, 2010; Barat and Shogan, 2025; Keikhosravani et al., 2025). Under these conditions, planktonic bacteria adhere and survive more readily, producing consistent infection at inoculum levels. At implantation, the implant surface is unencapsulated, and local innate defenses are acutely disrupted, strongly favoring bacterial adhesion (“race for the surface”). By 4 weeks, peri-implant fibrous encapsulation and an established local immune milieu create physical and immunological barriers. This maturation explains the substantially higher inoculum required in the delayed setting, consistent with the concept of a critical bacterial threshold that must be exceeded to disturb the established host–implant equilibrium (Dong et al., 2022; Shiels et al., 2020; Zhang et al., 2023). By 4 weeks, wound closure, peri-implant tissue/callus, and a localized immune milieu create physical and immunological barriers (Daghighi et al., 2013; Grzeskowiak et al., 2020; Wang et al., 2023; Zhang and Jin, 2024). Consistent with this, only $\geq \;10^{8}$ CFU per rat reliably overcame defenses in our model. Overall, infection risk is highest at implantation; after uncomplicated healing, a much higher challenge is required to disrupt the host–implant equilibrium (Cabrera et al., 2023; Chang and Lee, 2011; Gottenbos et al., 2001; Lex et al., 2022).

Beyond timing, phenotype matters in the delayed setting. Biofilm-derived aggregates (D-B-10^8^) yielded higher implant-associated burdens than planktonic inocula (D-P-10^8^), with limited differences in surrounding bone. This aligns with the enhanced infectious potential of biofilm fragments (Grari et al., 2025; Mishra et al., 2024). EPS shields bacteria from immediate clearance and promotes surface adhesion (Fujimura et al., 2015), and biofilm physiology alters surface/virulence profiles that aid attachment and immune evasion (Meganathan et al., 2022; Singhal et al., 2019), thereby facilitating infection establishment (Khatoon et al., 2018). Clinically, although hematogenous seeding often begins with planktonic cells, biofilm shedding can re-colonize implants and sustain chronicity (Han et al., 2016; Rao, 2020; Wang et al., 2017; Zhang et al., 2022). Body weight changes were consistent with infectious burden as a descriptive trend, while controls and lower-dose delayed groups maintained or gained weight (Campisi et al., 2002; Njiké Ngamga et al., 2021; Zhao et al., 2023).

Notably, rapid biofilm formation is expected after adhesion in both windows; our comparisons therefore primarily reflect challenge timing bacterial state and peri-implant maturity, not the mere presence/absence of biofilm.

Despite the promising development of this infection model, there are limitations to acknowledge. First, while our results demonstrated clear trends, the limited number of animals means statistical power is low, and findings should be interpreted as indicative of feasibility rather than validated reproducibility. Second, the requirement of 10^8^ CFU to establish delayed infection is substantially higher than typical hematogenous bacterial loads. Importantly, no animals in the 10^8^ CFU groups (D-P-10^8^ and D-B-10^8^) exhibited signs of systemic illness during the 14 d post-inoculation period; body weight loss remained within - 2 % to - 5 %, and no animals reached predefined humane endpoints for systemic sepsis, suggesting that infection remained localized to the implant site rather than progressing systemically. Nevertheless, whether this inoculum level models late surgical contamination, hematogenous seeding, or a stress-testing paradigm of host defense remains an open question that should be addressed in future work. Third, micro-CT was used qualitatively only, to confirm hardware positioning and implant integrity. Quantitative bone parameters, including bone volume, osteolysis, and peri-implant changes, were not analyzed, as the primary endpoint was bacteriological. Future studies should incorporate quantitative micro-CT endpoints to capture peri-implant bone changes associated with infection and to strengthen the imaging readout of the model. Fourth, our confirmation of infection relied on microbiological cultures and qualitative imaging, without accompanying histological analyses. Future studies should add histopathology to verify bacterial localization and biofilm architecture, especially in delayed-biofilm groups. Delayed delivery can vary with percutaneous injection (Experiment 2); surgical re-exposure in Experiment 3 likely improved placement but may transiently weaken local barriers. Standardizing delayed delivery (e.g., ultrasound-guided placement) should reduce variability across laboratories.

Looking ahead, our study provides a platform that addresses a specific gap: evaluating antibacterial implant coatings under two distinct host–implant maturation states within a single standardized construct, a feature not fully addressed by simpler intramedullary or non-segmental models. The small group sizes reflect the exploratory, model-establishment nature of this work, designed to identify conditions warranting larger-scale validation and consistent with the 3Rs of minimizing animal use in pilot studies (replacement, reduction, and refinement). More specifically, with further refinement and larger-scale validation, this dual-time-point inoculated model can serve as a useful platform for improving preventative and therapeutic strategies against implant-associated bone infections, ultimately helping to safeguard patients receiving orthopedic implants.

## 5 Conclusions

We established and optimized a rat femoral-defect model that reproduces immediate perioperative and delayed post-implantation contamination at the implant site. Early inoculation with 10^4^ to 10^6^ CFU per rat generated consistent early infections, whereas delayed infection 4 weeks after surgery required a higher challenge dose of 10^8^ CFU, with biofilm-derived inoculation producing the highest implant-associated bacterial burdens. This dual-time-point inoculated model offers clinically relevant CFU readouts and provides a practical platform for evaluating antibacterial coatings and other anti-infective strategies under both early- and delayed-infection conditions within a single, standardized framework.

## Data Availability

The datasets generated and analyzed in this study are available in Zenodo at 10.5281/zenodo.22009740 (Keikhosravani, 2026).

## Code availability

No custom code was generated in this study. Image analysis was performed using ImageJ v1.48 (https://imagej.net) with the BoneJ plugin v1.3.12 (https://bonej.org), both freely available.
